# Functional Annotation of Genetic Loci Associated With Sepsis Prioritizes Immune and Endothelial Cell Pathways

**DOI:** 10.3389/fimmu.2019.01949

**Published:** 2019-08-14

**Authors:** Kieu T. T. Le, Vasiliki Matzaraki, Mihai G. Netea, Cisca Wijmenga, Jill Moser, Vinod Kumar

**Affiliations:** ^1^University of Groningen, University Medical Center Groningen, Genetics Department, Groningen, Netherlands; ^2^Department of Internal Medicine and Radboud Centre for Infectious Diseases, Radboud University Medical Center, Nijmegen, Netherlands; ^3^Department of Immunology, K.G. Jebsen Coeliac Disease Research Centre, University of Oslo, Oslo, Norway; ^4^University of Groningen, University Medical Center Groningen, Department of Critical Care and Department of Pathology and Medical Biology, Groningen, Netherlands

**Keywords:** sepsis GWAS, cytokine QTLs, eQTL, functional genomics, PBMC transcriptome, endothelial response, FER locus

## Abstract

Due to limited sepsis patient cohort size and extreme heterogeneity, only one significant locus and suggestive associations at several independent loci were implicated by three genome-wide association studies. However, genes from such suggestive loci may also provide crucial information to unravel genetic mechanisms that determine sepsis heterogeneity. Therefore, in this study, we made use of integrative approaches to prioritize genes and pathways affected by sepsis associated genetic variants. By integrating expression quantitative trait loci (eQTL) results from the largest whole-blood eQTL database, cytokine QTLs from pathogen-stimulated peripheral blood mononuclear cells (PBMCs), publicly available blood transcriptome data from pneumoniae-derived sepsis patients, and transcriptome data from pathogen-stimulated PBMCs, we identified 55 potential genes affected by 39 independent loci. By performing pathway enrichment analysis at these loci we found enrichment of genes for adherences-junction pathway. Finally, we investigated the functional role of the only one GWAS significant SNP rs4957796 on sepsis survival in altering transcription factor binding affinity in monocytes and endothelial cells. We also found that transient deficiency of *FER* and *MAN2A1* affect endothelial response to stimulation, indicating that both *FER* and *MAN2A1* could be the causal genes at this locus. Taken together, our study suggests that in addition to immune pathways, genetic variants may also affect non-immune related pathways.

## Introduction

Sepsis is a major global health problem primarily caused by bacterial and fungal infections. It is a life-threatening organ dysfunction characterized by a dysregulated host immune response (1). The global burden of sepsis is high, with an estimated worldwide incidence of more than 30 million cases per year leading to nearly 6 million annual deaths (2). Regretfully, current strategies using a “one-size-fits-all” treatment approach for sepsis have failed because of the extreme heterogeneity in disease outcome (3). It is becoming increasingly clear that the heterogeneity is determined by impact of multiple risk factors including host genetic variation and pathogens (2). Therefore, identifying the critical genetic factors that affect sepsis patient outcome will help us to unravel genetic mechanisms that determine sepsis heterogeneity.

Up to now, three genome-wide association studies (GWAS) have been conducted to identify risk genes for sepsis. Two GWAS were conducted to identify associations between single nucleotide polymorphisms (SNPs) and 28 day sepsis mortality (4, 5). Another GWAS was conducted in a cohort of extremely premature infants to identify genetic loci associated with sepsis onset (6). However, only one study identified a genome-wide significant association at non-coding SNPs in the intron of Fps/Fes related tyrosine kinase (*FER*) gene in patients with 28 day survival of sepsis due to pneumonia (4). Although, these studies identified associations with several common polymorphisms, it is unclear how these SNPs affect sepsis outcome. Moreover, which genes and pathways in these loci affect sepsis survival remains to be studied. Identifying these specific genes and pathways is crucial to better understand the molecular mechanisms underlying sepsis heterogeneity.

System genetic approaches have been very effective for many complex human diseases, to translate genetic associations into functional understanding (7). By integrating multiple molecular phenotypes such as gene expression, protein levels, metabolites etc. with SNPs that were associated with human diseases, studies have shown that it is possible to prioritize potential causal genes affected by GWAS SNPs and obtain insights into functional pathways that affect human disease (8). Given the polygenic nature of many complex phenotypes, SNPs that are associated with suggestive significance also provide crucial biological insights. Moreover, as GWAS SNPs function in cell-type and context-dependent manner (9), integrating such context-specific molecular data with sepsis-associated SNPs may be more effective to obtain mechanistic insights into sepsis heterogeneity.

Therefore, in this study, we used pathogen- and cell-type specific gene expression levels, cytokine responses, and genotype data from population-based cohorts to integrate molecular responses with sepsis associated SNPs. We show that about 35% of the SNPs affect gene expression (eQTLs) in blood and <30% of sepsis associated SNPs affect cytokine production by peripheral blood mononuclear cells (PBMCs) in response to pathogens. Next, we show that the genome-wide significant SNP rs4957796 in the *FER* locus affects transcription factor binding efficiency in both monocytes and endothelial cells, and *FER* and (Mannosidase Alpha Class 2A Member 1 *(MAN2A1*) *MAN2A1* could be the causal genes in this locus via regulating endothelial function.

Taken together, our study provides evidence for genetically determined variability in endothelial pathways, in addition to leukocyte responses, as one of the important factors to explain sepsis heterogeneity. Therefore, more studies on the effect of the SNPs on different pathways such as barrier function or endothelial function are needed.

## Materials and Methods

### Identification of Proxy SNPs

Two hundred eighteen proxy SNPs from 39 independent loci were extracted from Haploreg using D′ = 1, *R*^2^ ≥ 0.95 using 1,000 Genome CEU as a reference population.

### Integration of Suggestive GWAS Loci With eQTL Data and Cytokine QTL Data

We made use of published eQTL data from eQTLgen (http://www.eqtlgen.org) and in house-cytokine QTL from 500 FG (10). We extracted only genome-wide significant eQTL signals from eQTLgen. Briefly, cis-eQTL analysis was performed in 31,684 blood samples. Significant cis-eQTLs were defined as eQTLs that show FDR corrected *P*-value of <0.05 (*P* < 1,829 × 10^−5^) (11). For cytokine QTLs from the 500 FG cohort study (cytokine QTLs were performed on 392 individuals), we extracted reported *P*-value for each SNP (10) and applied Bonferroni correction to define significant *P*-value threshold. As we tested 39 independent loci, the *P*-value threshold is 0.0012.

### PBMC Transcriptome

We made use of in house PBMC transcriptome data (12). Briefly, PBMCs were freshly isolated from peripheral venous blood withdrawn from healthy volunteers, according to work permission on whole blood (Ethical Committee of Radboud University Nijmegen (nr 42561.091.12). PBMC were freshly isolated with Ficoll-Paque (Pharmacia Biotech), counted (BioRad cell counter), and adjusted to 5 million cells/ml in RPMI 1640 (Gibco, ThermoFisher Scientific), supplemented gentamicin 10 mg/ml, L-glutamine 10 mM, and pyruvate 10 mM. Cells were seeded into wells to settle overnight before stimulation. PBMC were stimulated with 100 μl of *Streptococcus pneumonia* (ATCC 49619, serotype 19F) (1 million cells/ml), *Candida albicans* (ATCC MYA-3573, UC 820) (1 million cells/ml), *and Pseudomonas aeruginosa* (1 million cells/ml). PBMCs were also stimulated with RPMI a negative control. RNA was isolated at 4 and 24 h after stimulation. RNA sequencing was performed in a Nextseq 500 platform, single-end, read length 50 bp. Reads were mapped to the human genome hg19 using STAR (version 2.3.0), Ht-seq count was used to quantify read counts and DEseq2 was used to perform statistics analysis (FDR *P* ≤ 0.05 and fold change ≥2) (12).

### Gene Expression in Pneumoniae-Derived Sepsis Patients

To further validate our prioritized genes, we checked their expression levels in pneumoniae-derived sepsis patient transcriptome (*N* = 265 patients) (13). Briefly, we extracted only genome-wide significant differentially expressed genes (FC>1,5, and FDR correct *P*-value of 0.05) reported in either the discovery cohort or the validation cohort (13).

### Electrophoresis Mobility Shift Assay (EMSA)

EMSA were performed using LightShift Chemiluminescent EMSA Kit (Thermo Scientific) according to the manual instruction. In brief, the protocols contain three main parts, including: probe biotination, nuclei extraction, and mobility shift assay on polyacrylamide gel. Probe biotination. Probes containing nucleotide sequence of 30 bp around the SNP were designed carrying either T allele or C allele at the SNP position. The probes were then labeled with biotin at the 3′ end using Pierce Biotin 3′ End DNA labeling kit (Thermo Scientific). After labeled, probes were annealed to make double stranded DNA probes. Labeling efficiency was evaluated following the recommended protocol. Nuclei extraction. Ten million cells were used to isolate the nuclei. Cells were suspended in lysis buffer (10 mM Tris-Cl pH8.0, 300 mM sucrose, 10 mM NaCl, 2 mM MgAc2, 6 mM CaCl2, and 0.2% of NP-40 (Igepal) for 5 min. Nuclei pellets were harvested and resuspended in 100 μl of Nuclear Extract Buffer (20 mM Tris-Cl (pH8.0), 420 mM NaCl, 1.5 mM MgCl2, 0.2 mMEDTA, 25% glycerol, 1 mM DTT and 1X protease inhibitor cocktail) for 10 min on ice. After centrifugation at 14.000 rpm for 15 min, supernatant containing nuclear extract was collected and protein concentration was determined using Bradford assay. Gel mobility assay. Mobility assay was performed according to the instruction. Briefly, 5–10 μg of total proteins from the nuclei extract was used with 20 fmol of Biotin End-labeled target DNA. Unlabeled target DNA was also used as a binding competition in the presence or absence of protein from nuclei extract. Images were obtained using BioRad system.

### Cell Culture

To mimic the context of sepsis in which inflammation involves the role of endothelial cells and blood cells, we used Primary Human Umbilical Vein Endothelial cells (HUVEC) (Lonza, The Netherlands) as endothelial cells and THP-1 (ATCC, The Netherlands) as monocytes. Pooled donor-HUVEC were purchased from Lonza (C2519A, The Netherlands). Cells were cultured in EBM-2TM medium (Lonza) supplemented with EGM-2 MV SingleQuot Kit Supplements & Growth Factors (Cat. No. 3202, Lonza) and antibiotics 100 IU/ml of penicillin (Astellas Pharma, The Netherlands) and 50 μg/ml of Streptomycin (Rotexmedica GmbH, Germany). Cells were used from passage 3–7 and cultured at 37°C, 5% CO2 and saturating humidity. THP-1 cells (ATCC, The Netherlands) were cultured in Gibco TM RPMI 1640 containing L-glutamine +/HEPES + (Cat. No. 1640 52400-025) supplemented with 10% (v/v) heat-inactivated FBS (Gibco), 1%(v/v) Pen/Strep 10.000 U/ml (Gibco). THP-1 cells were kept at 37°C, 5% CO2 and saturating humidity. Cells were freshly passed twice a week to keep a density of 200.000–800.000 cells/ml and used up to passage 28.

### Knock Down Experiment in HUVEC

HUVEC were seeded to reach the confluency of 70% before transfection. siFER and siMAN2A1 were delivered into HUVEC by Lipofectamine 2000 (Invitrogen). 20 pmol of siRNA sequence was transfected into one million cells according to instructed protocol. After transfection, cells were rested for 48 h before subsequent stimulation with LPS derived from *Escherichia coli*, serotype O26:B6 (15,000 endotoxin units/g) (Sigma-Aldrich, St.Louis) (1 μg/ml) (14). Cells were lysed in Trizol (Ambion, ThermoFisher) and kept at −80°C until RNA isolation.

### Gene Expression by RT-qPCR

Gene expression levels were measured by RT-qPCR (reverse transcriptase-quantitative PCR) using Sybrgreen platform. Briefly, total RNA was isolated by Trizol according to the instructed protocol. RNA concentration was measured by Nanodrop. RNA quality was controlled in random samples by measuring RNA Integrity Score (Agilent). 100–5,000 ng of total RNA was loaded for cDNA synthesis using ReverAid H Minus First Strand cDNA synthesis kit (ThermoScientific). Primers (refer to Table 1) were designed with primer3 and conditions were optimized for each primer set. Melting curves were used to access the specificity of each reaction. GAPDH was used as a housekeeping gene. qPCR was performed in a ViiA7 real-time PCR (Applied Biosystems) following the standard protocol: 15 min at 95°C and 40 cycles of two steps: amplification (60°C for 60 s) and denaturation (95°C for 15 s). Gene expression levels were calculated based on the comparison of CT values between target gene(s) and the housekeeping gene (ΔCT). Average messenger RNA levels relative to GAPDH from the duplicate were calculated by 2^−Δ*CT*^ Data were shown as mean ± SD. One-way ANOVA test was used to compare between conditions and control: *P* ≤ 0.05 (^*^); *P* ≤ 0.01 (^**^); *P* ≤ 0.001 (^***^); *P* ≤ 0.0001 (^****^). GraphPad Prism software (version 6.0) was used to make graphs and determine significant differences.

**Table 1:** Primer sequences.

**Primer**	**Sequence (5^**′**^-3^**′**^)**
MAN2A1_forward	CGCAGAAAATGATACACACGG
MAN2A1_reverse	CGTGGCTCTTTCCTAAACAGG
GAPDH_forward	CTGCATTTCATTCCAGTTCAGG
GAPDH_reverse	TCTGTCCAGTGATTCAGCCA
FER_forward	CAAATCAGCAAGCAAGAGAGC
FER_reverse	TGAACTTAGGGCGATTTTCAGG
ICAM1_forward	GGCCGGCCAGCTTATACAC
ICAM1_reverse	TAGACACTTGAGCTCGGGCA
VCAM1_forward	TCAGATTGGAGACTCAGTCATGT
VCAM1_reverse	ACTCCTCACCTTCCCGCTC
Eselectin_forward	CCCGAAGGGTTTGGTGAG
Eselectin_reverse	TAAAGCCCTCATTGCATTGA
IL8_forward	TCTGCAGCTCTGTGTGAAGG
IL8_reverse	ACTTCTCCACAACCCTCTGC
Probe_Sense (T)	CAAAATTTATAAATAT**T**ACATCATTGAAATTAT
Probe_Antisense (T)	ATAATTTCAATGATGT**A**ATATTTATAAATTTTG
Probe_Sense (C)	CAAAATTTATAAATAT**C**ACATCATTGAAATTAT
Probe_Antisense (C)	ATAATTTCAATGATGT**G**ATATTTATAAATTTTG

## Results

### Annotation of 39 Independent Loci From Three Sepsis GWAS

Two genetic studies were conducted to identify SNPs associated with sepsis survival in adult patients (28-day mortality) and one study on sepsis onset in extremely premature infants. We extracted 25 SNPs that are associated with sepsis survival with evidence for suggestive association (*P* < 10^−5^), which includes 11 SNPs from Rautanen et al., and 14 SNPs from Scherag et al. study (4, 5). Using the same criteria we extracted 30 SNPs that are associated with sepsis onset in infants from Srinivasan et al. study [(6); Table S1]. Among these 55 SNPs, we filtered by locus position, for loci located within 1 Mb from each other, and selected a SNP with the lowest P-value as the representative. As a result, we found 39 independent loci from the three GWAS. We then extracted 218 proxy SNPs (*R*^2^ ≥ 0.95, D′ = 1) for these 39 independent SNPs using 1000 Genome CEU as a reference population (Table S1). As previously reported, none of these loci were shared between the three studies. Although, this may be because of the insufficient study power, it also emphasizes the clinical heterogeneity among patients between cohorts, which could be partly determined by genetic variations. Therefore, we followed up these independent loci to prioritize potential causal candidate genes and pathways affected by them.

### Expression QTL Mapping and Differential Expression Analyses Prioritized Potential Causal Pathways for Sepsis

To identify potential causal genes affected by sepsis-associated SNPs, we made use of expression-QTL (eQTL) analysis. For this we extracted results from the largest eQTL study (eQTLGen) that included nearly 35,000 blood samples (11). We found significant association of SNPs from 13 independent loci with expression levels of 45 unique genes (Table 2). Interestingly, three loci that were associated with sepsis onset in extremely premature infants affected the most number of nearby genes (Table 2). In particular, SNPs rs12490944, rs41461846, and rs3844280 affected 14, 10, and 5 genes, respectively.

**Table 2:** Summary table of genes and cytokines of which the expression levels are associated with genetic variations at 39 GWAS suggestive loci. 13/39 loci could alter RNA expression level of 45 nearby genes (cis-eQTL).

**Study**	**Independent loci**	**cis-eQTL (blood)**	**eQTL-P value**	**Cytokine QTL**	**cQTL-P value**
Rautanen A	rs2709532	No		No	
	rs72661895	No		No	
	rs4957796	No		No	
	rs79423885	No		No	
	rs76881522	No		No	
	rs12114790	CSGALNACT1^a^	9.50E-66	IL1b_C.albicansconidia_PBMC_24h	0.010228723
		INTS10	3.27E-09	IL6_C.albicanshyphae_PBMC_24h	0.026800224
				TNFA_C.albicansconidia_PBMC_24h	0.040662939
	rs9566343	No		IL22_C.albicansconidia_PBMC_7days	0.009406105
				IL6_LPS100ng_PBMC_24h	0.022034182
	rs6501341	No		No	
	rs2096460	URB1^a^	6.89E-152	No	
		C21orf119	1.57E-21		
Scherag A	rs382422	WLS^b^	8.66E-12	IFNy_C.albicansconidia_PBMC_7days	0.006355623
	rs150811371	No		No	
	rs945177	No		No	
	rs9529561	No		No	
	rs2641697	CRISPLD2^a, b^	1.18E-08	IL6_S.aureus_PBMC_24h	0.029215619
		KIAA0513^b^	6.31E-07		
	rs7211184	No		No	
	rs58764888	No		No	
	rs72862231	No		No	
	rs150062338	No		No	
	rs10933728	No		No	
	rs115550031	DGKQ^a^	5.95E-06	No	No
	rs62369989	No		IL17_C.albicansconidia_PBMC_7days	0.011402725
	rs117983287	No		No	
	rs409443	No		No	
Srinivasan L	rs3100127	PTPN7	3.48E-91	No	
		LGR6^b^	6.01E-16		
	rs41461846	CYP27A1^b^	3,2717E-310	No	
		RQCD1	3,2717E-310		
		VIL1^a^	4.3769E-101		
		TTLL4^a^	1.3023E-79		
		STK36	9.0469E-76		
		USP37^a^	4.9084E-71		
		SLC11A1^a, b^	3.4111E-61		
		ZNF142	2.6409E-55		
		PRKAG3^b^	6.0805E-38		
		BCS1L	1.8409E-37		
	rs72998754	No		No	
	rs3844280	BRK1^a^	1.83E-180	No	
		LINC00852	6.43E-19		
		FANCD2^a^	8.65E-12		
		IRAK2^a, b^	6.97E-11		
		CRELD1	5.51E-06		
	rs12490944	RBM6^a^	2.01E-195	No	
		HYAL3^b^	2.98E-98		
		MON1A^a^	1.56E-79		
		UBA7^b^	5.21E-59		
		APEH	5.64E-27		
		AMT	3.54E-21		
		NICN1	2.61E-20		
		IFRD2	4.12E-10		
		NAT6	2.39E-08		
		KLHDC8B^b^	4.60E-08		
		QRICH1	2.34E-07		
		TCTA	1.42E-05		
		MST1^b^	1.57E-05		
		FAM212A	1.74E-05		
	rs17599816	No		No	
	rs6462728	AOAH	1.65E-26	IL17_C.albicansconidia_PBMC_7days	0.010838542
				IL6_C.albicansconidia_PBMC_24h	0.020869368
	rs2237499	LINC00265	4.59E-91	IL1b_LPS100ng_PBMC_24h	0.000626831
		RALA^a^	5.32E-18	TNFA_C.albicansconidia_PBMC_24h	0.012141737
		CDK13	7.48E-14	IL6_LPS100ng_PBMC_24h	0.017978737
				IL1b_E.Coli_PBMC_24h	0.033518435
	rs4730486	IMMP2L	3,2717E-310	No	
	rs513793	No		No	
	rs11597285	No		No	
	rs74487835	No		No	
	rs16913666	No		No	
	rs11840143	No		IL22_C.albicansconidia_PBMC_7days	0.021643788
				IFNy_C.albicansconidia_PBMC_7days	0.049325944
	rs13380717	No		IFNy_C.albicanshyphae_PBMC_7days	2.51E-06
				IL22_C.albicanshyphae_PBMC_7days	0.003182544
				TNFA_E.Coli_PBMC_24h	0.032629607
				IL1b_E.Coli_PBMC_24h	0.043946978
	rs645505	NAPG	5.33E-06	No	

a Gene locates within 200 kb window surrounding the suggestive GWAS loci.

b *eQTL genes of which RNA expression levels are differentially expressed in stimulated PBMCs. 9/39 loci could alter cytokine levels upon stimulation (cytokine-QTL)*.

**Figure 1:**
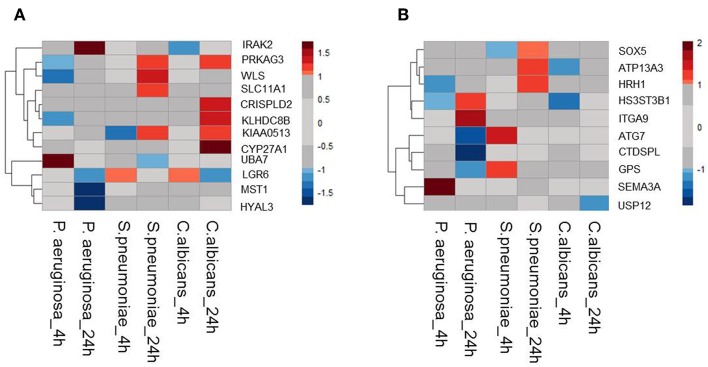
Expression QTL mapping and differential expression analyses prioritized potential genes. **(A)** Among 45eQTL genes, there are 12 genes that are differentially expressed in at least one condition in stimulated PBMCs. **(B)** Expression levels of cis genes that have not eQTL effect in blood, but differentially expressed upon stimulation in PBMC. Heatmap was plotted based on log2 (Fold-change) of RNA expression levels in *P.aeruginosa, S.pneumoniae*, and *C.albicans*-stimulated PBMCs. RNA expression levels were measured after 4 or 24 h of stimulation. Colors represent the RNA expression levels, red, significantly induced genes; blue, significantly suppressed genes; gray, not significantly different between stimulated and non-stimulated PBMCs.

Moreover, it is shown that differentially expressed genes in response to infectious agents are more likely to be associated with susceptibility to infectious diseases (15) and more than 90% of the lead SNPs that have eQTL effects are located within 100 kb of the eQTL genes (11). Therefore, as a second strategy to prioritize potential causal genes at sepsis-associated loci, we tested the expression levels of all genes located within a 200 kb window of all 39 loci with suggestive association (*P* < 9.99 × 10^−5^) in stimulated peripheral blood mononuclear cells (PBMCs) transcriptome. For this, we used RNAseq data from PBMCs that were stimulated with *Pseudomonas aeruginosa* (*P. aeruginosa*), *Streptococcus pneumoniae* (*S. pneumoniae*) or *Candida albicans* (*C. albicans*) for 4 or 24 h. We found that 12 out of 45 *cis*-eQTL genes (26,67%) were also differentially expressed in at least 1 condition (Figure 1A). In addition, we also found another 10 *cis*-genes, which were not implicated by eQTL mapping as causal genes, to be differentially expressed in at least one of the stimulations in PBMC (Figure 1B). In the end, by combining these two strategies, we prioritized 55 potential causal genes for sepsis.

### Subsets of Prioritized Genes Are Also Associated With Severity of Sepsis

**Figure 2:**
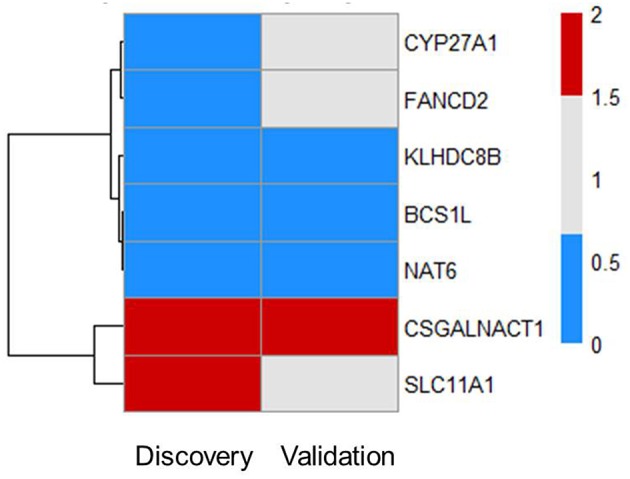
Subsets of prioritized genes are also associated with severity of sepsis. Among 55 genes, there are seven genes that are DE in patients (FC > 1.5 and FDR ≤ 0.05). Heat map shows RNA expression levels of seven genes in both discovery and validation cohort. Colors represent expression levels by fold-change between two groups: severe patients SR1 vs. mild patients SR2. Blue, significantly lowly expressed in the severe group; red, significantly highly expressed in the severe group; white, non-significantly different between the severe and mild groups (13).

Next, we tested whether some of the prioritized sepsis-associated genes show any correlation with the severity of sepsis. To perform this analysis, we made use of publicly available blood transcriptome data from pneumoniae-derived sepsis patients (13). Out of 55 prioritized genes, we found seven genes that are differentially expressed between severe and mild sepsis patients (Figure 2). Among them, expression of *CSGALNACT1* is increased in severe patient group whereas *KLHDC8B, BCS1L*, and *NAT6* expression levels were decreased. Interestingly, except *CSGALNACT1*, all the other six genes were eQTL genes for SNPs associated with sepsis onset. This observation suggests that some of the genes associated with disease onset could also be involved in determining disease severity.

There was no evidence for enrichment of these six genes for particular pathways; however, *CYP27A1* and *SLC11A1* are known to be involved in sepsis. CYP27A1 is one of the key enzymes involved in synthesizing bile acid in the liver. Studies have shown that CYP27A1 down regulation in sepsis reduce the amount of circulating bile acid, which may be beneficial for sepsis patients (16, 17). *SLC11A1* encodes for iron channel, involved in cation metabolism and host resistance to infection. SLC11A1 was shown to be associated with active tuberculosis (18–20). It remains to be tested how these genes contribute to sepsis severity.

### Around 23% of the Loci Affect Cytokine Production by Leukocytes in Response to Sepsis Causing Pathogens

**Figure 3:**
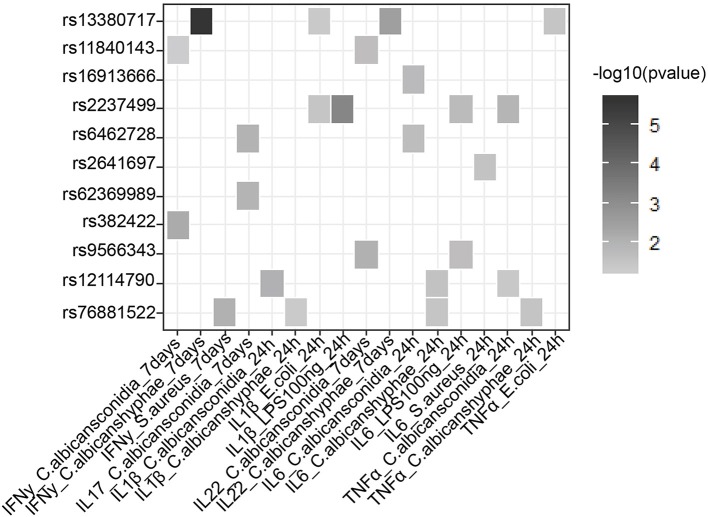
Suggestive GWAS loci could influence the production of cytokines from PBMC in response to infection. Heat map shows cytokine- QTL (cQTL) effect of the suggestive SNPs (*P*-value ≤0.05), based on 500 FG cytokine QTL data (10). Empty boxes indicate no cQTL relationship between the SNPs and cytokine production. Color darkness was scaled base on –log10 (*P*-value).

In addition to a global screening for the effect of 39 suggestive loci on transcriptome response, we also tested their effects in regulating inflammatory cytokine responses, a prominent phenotype in sepsis. We tested if SNPs that are associated with sepsis survival or sepsis onset affect production of cytokines by leukocytes upon stimulation by intersecting our 218 SNPs with cytokine QTL from stimulated PBMCs (10). We found that 9 independent loci affect the production and secretion of six different cytokines in the context of Gram-negative bacteria, Gram- positive bacteria and fungi (Table 2 and Figure 3), albeit with nominal statistical significance (*P* < 0.05). Only two loci, among these 9 loci, are found to be significantly associated with cytokine production in PBMCs after correcting for multiple testing (P < 0.0012) (Table 1). In particular, SNP rs2237499 affected IL-1β levels upon LPS (Gram-negative bacterial infection), whereas SNP rs13380717 altered IFN-γ levels in response to *C. albicans* hyphae infection. In summary, only around 23% of the sepsis-associated variants affected cytokine production. These results suggest that the other non-cytokine processes are also important for explaining sepsis heterogeneity.

### Sepsis Associated Genes Are Enriched for Adherence Junction Pathway

**Figure 4:**
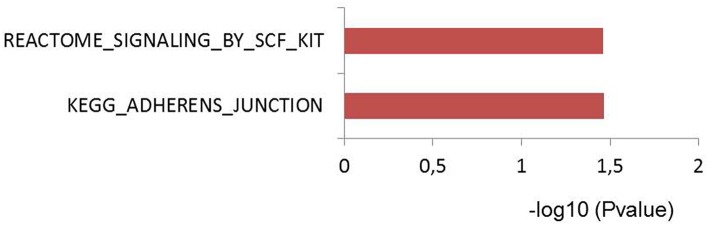
Pascal pathway enrichment for 39 independent suggestive-GWAS loci (21). Y axis: pathways enriched by Reactome and KEGG database. X axis: –log(10) of *q*-value.

To test if genes affected by sepsis survival associated SNPs are enriched for particular biological pathways, we made use of Pascal pathway prioritization tool (21). Based on the SNP location, and the *P*-value of each SNP, the Pascal software will calculate gene score of nearby genes, and the probability of each gene in involving in any signaling pathways. We initially performed gene prioritization and pathway enrichment analyses for each study separately. However, because of less number of loci from each study, we were unable to see strong enrichment of any pathways. We, therefore, combined all 39 independent loci from three studies and performed enrichment analysis. Interestingly, the enrichment analysis showed significant enrichment of genes for adherences-junction pathway (Figure 4).

Particularly, the enrichment analysis was based on 36 genes located within 100 kb of 39 independent SNPs. Among those, there are 17 genes that overlapped with the 55 prioritized genes above (data not shown). These findings strengthen the common notion that disruption in barrier, especially vascular wall leakage is a critical process, which lead to organ dysfunction and mortality in sepsis.

### Regulatory Function of GWAS SNP rs4957796 at FER Locus in Endothelial Cells

We showed that many of the sepsis associated SNPs affect gene expression or alter cytokine levels in response to infections in blood. However, we didn't find any association with expression or with cytokine responses for SNP rs4957796, which is the only genome-wide significant SNP from a GWAS, at FER locus (Table 2). This SNP is associated with the survival of pneumonia-derived septic patients. However, how the SNP contributes to the disease severity or which genes are affected by this SNP is not clearly established. Therefore, we conducted experiments in both immune cells and endothelial cells (HUVEC), which play central roles in sepsis pathogenesis (22).

**Figure 5:**
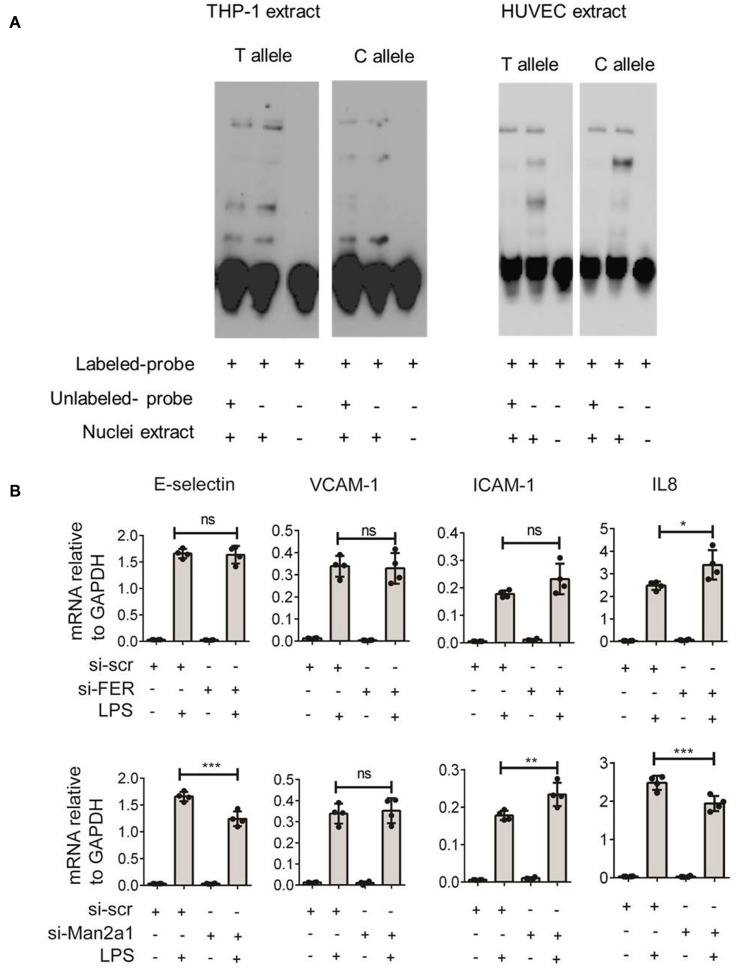
Validation of rs4957796 SNP. **(A)** EMSA (electrophoresis molecular shift assay) of oligos resembling the sequence of 30 nts surrounding the top SNP: rs4957796, containing either T or C allele. The shift in the position of the probe carrying T or C allele indicated the effect of nucleotide alteration at rs4957796 in changing the binding affinity of transcription factor. **(B)** Effect of *FER* or *MAN2A1* deficiency in HUVEC on the expression of adhesion molecules and cytokines. RNA expression levels of E-selectin, VCAM-1, ICAM-1, and IL-8 in HUVEC after 4 h of stimulation were measured by RT-qPCR. Each dot represents one sample. Data represent for three replications.

To gain further insight into the function of this SNP, we tested if the SNP could alter the binding site of transcription factors. The alteration of nucleotide composition can lead to changes in the binding of these transcription factors, hence, affecting expression levels of genes. Based on weight matrix prediction (23) this SNP is located in the binding motif of several transcription factors (Figure S1). Next, we tested the expression of these transcription factors both in stimulated PBMCs and endothelial cells. We found that ARID5A, E4BP4, HLF, Jundm2, and Ncx_2 differentially expressed in PBMCs upon stimulation. On the other hand, these transcription factors (ARID5A, BBX, E4BP4, FOXL1, Jundm2, Mef2, TBP, and p300) were expressed in endothelial cells, yet the expression levels were not altered by stimulation of IL1β, TNFα, or LPS. Next, we performed electrophoresis molecular shift assay (EMSA) to validate if the SNP can alter binding affinities of transcription factors in endothelial cells (HUVEC) and monocytes (THP-1). We found that the alteration of T (the risk allele) to C allele (the alternative allele) resulted in changes in the competition of at least two transcription factors in binding to the locus (Figure 5A). The effects were shared between both cell types. These findings indicated that the genome-wide significant SNP at FER locus could alter the binding of transcription factors in endothelial cells as well as in monocytes to influence the expression of *cis*-genes. Therefore, future studies should generate large scale endothelial cell gene expression data upon relevant stimulations to establish the link between sepsis associated SNPs and cis-genes.

### Both FER and MAN2A1 Alter Endothelial Cell Responses to Stimulation

Previous studies have speculated that *FER* could be a potential causal gene at this locus (4). However, the expression levels of this gene in blood of sepsis patients did not show any correlation with the severity of sepsis (13). As SNPs can alter expression levels of multiple *cis*-genes, we tested if the expression of other nearby genes are associated with the disease severity using the data from Davenport et al. (13) and found *MAN2A1* to be differentially expressed between the two patient groups. We first stimulated endothelial cells with different infectious agents representative of Gram-negative bacterial antigen (LPS), Gram-positive bacteria (*Streptococcus pneumoniae*), and Fungus (*Candida albicans*). We observed strong activation of endothelial cells by LPS but not by other stimuli (Figure S2). We therefore focused only on LPS stimulation for knockdown experiments. We then performed transient knockdown experiments on both *FER* and *MAN2A1* genes in endothelial cells using gene-specific siRNAs. Interestingly, both *FER* and *MAN2A1* deficiency in HUVEC altered the cell response to LPS stimulation. We found that the knockdown of *MAN2A1* showed stronger effect on the expression of both adhesion molecules (E-selectin and ICAM-1) and cytokine genes (IL-8) (Figure 5B). Although, it is still needed to establish the connection between SNP and these two genes, these preliminary results highlight the role of more than one causal gene at this locus.

## Discussion

Host genetic variation is an important factor in explaining susceptibility to infectious diseases in general, and sepsis heterogeneity in particular. Up to now, three genome-wide association studies on sepsis have been conducted. However, due to limited sepsis patient cohort size and extreme heterogeneity, only one significant locus was identified by a GWAS. Nevertheless, the suggestive associations implicated by these three studies may provide novel insight into genes and pathways that are relevant for understanding sepsis heterogeneity.

In this study, we took advantage of existing molecular data and integrative functional genomics approach to reveal potential causal genes and pathways associated with sepsis heterogeneity. Firstly, we show that <30% of the sepsis associated loci affect cytokine production in response to pathogens. Some of these cytokine-affecting SNPs may be regulated via their effect on expression levels of its nearby genes (eQTL genes). For example, a *WLS* gene is located in cis-region of a SNP that affects IFN-γ production in PBMCs in response to Candida conidia (Table 1). In NK T cells, it is shown that the *WLS* gene can activate IFN-γ production independent of Wnt/B-catenin pathway (24). Another SNP that is associated with IL17 and IL6 levels upon Candida albicans conidida stimulation in PBMCs is close to *AOAH* gene (Table 2). *AOAH* codes for acyloxyacyl hydrolase that can deacylates and inactivate LPS, a toxin presented on Gram-negative bacteria wall. Studies have shown that *AOAH* can drive TH17 T cell differentiation via secreting IL-6 in mice (25). Therefore, it is likely that some of these genes may affect sepsis via regulating cytokine levels in response to infections.

On the other hand, it is possible that because of the lack of sufficient statistical power in these studies, some of these associations could be false positive findings. Nevertheless, it is interesting to observe that more than 70% of the loci were not correlated with cytokine levels suggesting the role of other functional pathways in sepsis. In concordance with this we also show that, by applying PASCAL gene prioritization tool, *cis*-genes are enriched for adherens junction pathway. However, pathway enrichment analysis on only eQTL genes did not reveal any pathways. It may be due to the fact that genetic effects on gene expression can be very tissue and stimulation specific (7). Therefore, the expression quantitative trait analysis in healthy blood samples may not reflect the effect of sepsis-associated genetic variants. More studies are needed to investigate the effect of genetic variants on different pathways such as coagulation, blood pressure, barrier dysfunction, and vascular leakage that are pivotal for sepsis pathogenesis. Our EMSA assays on a SNP located within FER locus also suggested that some of these sepsis associated SNPs may affect more than one causal genes. Therefore, these factors need to be taken into account when we establish causal genes from association studies. Nevertheless, eQTL mapping shows that 33% suggestive sepsis-associated loci can affect expression levels of 55 potential causal genes and some of these genes are differentially regulated in patients with severe sepsis compared to mild sepsis patient group. These genes are of interest to perform further functional studies to understand their role in sepsis onset and survival.

Our study also has several limitations. When we compared the sepsis associated SNPs from all three GWAS, we found that none of the SNPs were replicated in each other's study. This could be either due to the limited sample size and/or the extreme heterogeneity among sepsis patients caused by several factors including age of patients, type of infectious agents, clinical treatments etc. Therefore, in the future, a large-scale meta-analysis on stratified groups of sepsis patients should be done to identify genetic variations determining sepsis onset, sepsis severity or sepsis mortality. Moreover, to overcome the heterogeneity of sepsis, GWAS on sepsis-associated phenotypes such as vascular leakage, hypertension, organ damage will also be informative to gain further insights into sepsis endo-phenotypes. Secondly, eQTL mapping results were extracted only from whole blood of healthy individuals in this study. Given the prominent role of endothelial and other cell types in sepsis, future studies should focus on generating tissue and context-specific gene expression data to reveal causal genes for sepsis.

To conclude, our approach in this study provides evidence for genetically determined variability in endothelial pathways, in addition to leucocyte responses, as one of the important factors to explain sepsis heterogeneity. Future challenge is therefore to exploit the impact of genetic variation on endothelial cell related processes using both experimental and clinical studies, to develop new treatment options for sepsis.

## Data Availability

Publicly available datasets were analyzed in this study. This data can be found here: 500 FG cytokine: https://hfgp.bbmri.nl/, eQTL: http://www.eqtlgen.org/.

## Author Contributions

VK is accredited to the study conceptualization. KL, JM, and VK designed the study. KL performed experiments. KL and VM analyzed the data. MN, JM, and CW provided reagents, protocols, and facilities to conduct experiments. KL and VK prepared the manuscript. MN, CW, JM, and VK interpreted results and critically assessed the manuscript.

### Conflict of Interest Statement

The authors declare that the research was conducted in the absence of any commercial or financial relationships that could be construed as a potential conflict of interest.
