# Supplementary figures and images for: Functional Annotation of Genetic Loci Associated With Sepsis Prioritizes Immune and Endothelial Cell Pathways
Source: Front Immunol. 2019 Aug 14;10:1949. doi: 10.3389/fimmu.2019.01949 (PMC6703137; doi:10.3389/fimmu.2019.01949)

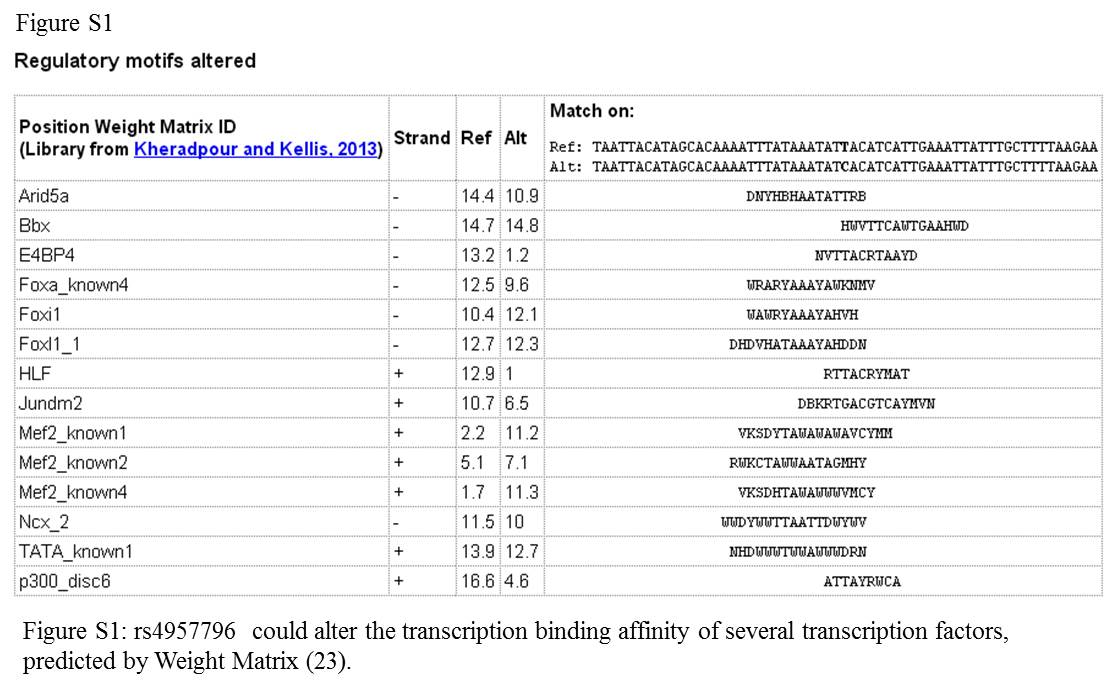

Supplement: Supplementary file 2 [file Image_1.JPEG]

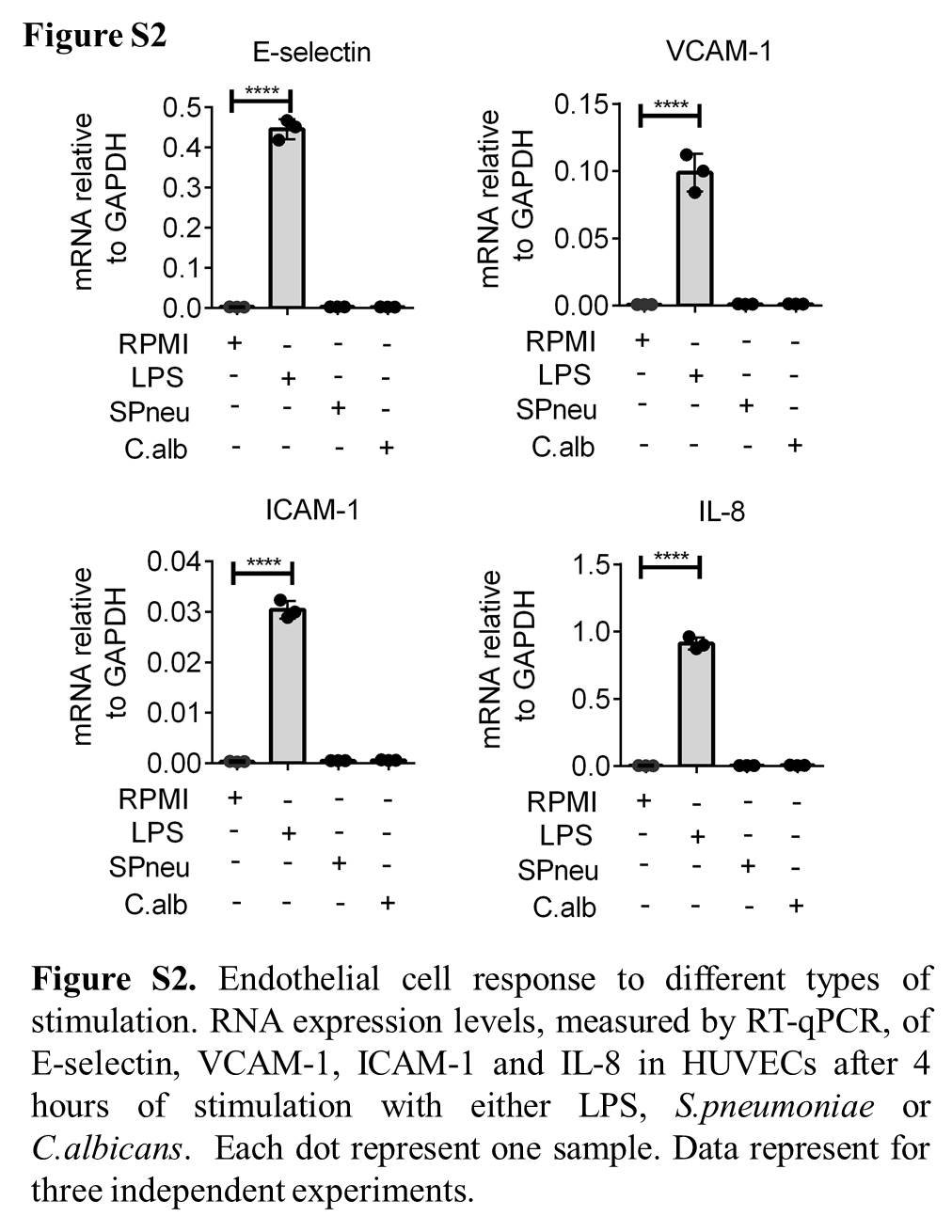

Supplement: Supplementary file 3 [file Image_2.jpg]
